# Compartmentalized gene expression profiling of receptive endometrium reveals progesterone regulated ENPP3 is differentially expressed and secreted in glycosylated form

**DOI:** 10.1038/srep33811

**Published:** 2016-09-26

**Authors:** Nageswara Rao Boggavarapu, Sujata Lalitkumar, Vijay Joshua, Sergo Kasvandik, Andres Salumets, Parameswaran Grace Lalitkumar, Kristina Gemzell-Danielsson

**Affiliations:** 1 Division of Obstetrics and Gynecology, Department of Women’s and Children’s Health, Karolinska Institutet/ Karolinska University Hospital, S-171 76, Stockholm, Sweden; 2 Competence Centre on Health Technologies, Tiigi 61b, 50410, Tartu, Estonia.; 3 Department of Obstetrics and Gynecology, University of Tartu, L. Puusepa 8, 51014, Tartu, Estonia.; 4 Department of Obstetrics and Gynecology, University of Helsinki and Helsinki University Hospital, Helsinki, Finland.

## Abstract

The complexity of endometrial receptivity at the molecular level needs to be explored in detail to improve the management of infertility. Here, differential expression of transcriptomes in receptive endometrial glands and stroma revealed Ectonucleotide Pyrophosphatase/Phosphodiesterase 3 (ENPP3) as a progesterone regulated factor and confirmed by various methods, both at mRNA and protein level. The involvement of ENPP3 in embryo attachment was tested in an *in vitro* model for human embryo implantation. Interestingly, there was high expression of ENPP3 mRNA in stroma but not protein. Presence of N-glycosylated ENPP3 in receptive phase uterine fluid in women confirms its regulation by progesterone and makes it possible to use in a non-invasive test of endometrial receptivity.

A significant cause of female infertility (10–15% globally) is the failure to establish a receptive endometrium, with the latter primarily driven by progesterone (P). Despite optimization of critical events in assisted reproductive technology, pregnancy rates do not exceed 30%^1^. Important limitations are the lack of knowledge about the implantation process, including endometrial receptivity and lack of reliable diagnosis of receptivity. Recent advances in omics technology, either by a genomic or proteomic approach, has yielded many molecules regulated during the receptive phase: integrins αvβ3^2^, LIF, gp130^3^, nuclear pore proteins^4^, HB-EGF^5^, mucins^6^, heart and neural crest derivatives 2^7^, homeobox genes^8^, Annexin A2^9^, Annexin IV^10^, Calreticulum^11^, Stathmin 1^9^, and Ezrin^12^, but no unequivocal receptivity marker is yet defined in humans.

Taking into consideration the dynamic nature of the endometrium, cellular and molecular signatures alter rapidly due to ovarian hormonal regulation in a given menstrual cycle^13^. Endometrial tissue sampling for identification of biomarkers in endometrial receptivity by microarray studies include divergent cohorts: normo-ovulatory women^14^,^15^,^16^,^17^,^18^,^19^,^20^,^21^,^22^, fertile donors^23^, fertile mid-secretory phase and infertile women^24^,^25^, natural and stimulated cycles^16^,^26^, and women with recurrent implantation failure or miscarriage^27^,^28^. The high degree of heterogenicity leads to difficulty in deriving conclusions for receptivity genes. Adding to this, the above studies were done using whole endometrial tissue, which constitutes diverse cell types in altered ratios with distinct genotypic and phenotypic expression, leading to different results.

The antiprogestin mifepristone administered on cycle day LH + 2 has previously been used to study P-regulated endometrial receptivity^29^,^30^,^31^. Potentially, this approach could also be used to identify a possible biomarker for endometrial receptivity. To overcome confounding factors such as tissue variability and to minimize subject-to-subject variations, we selectively isolated the mid-secretory phase glandular epithelium and stroma from fertile and infertile women by laser capture microdissection (LCMD), followed by microarray. The results were reconfirmed by real time PCR and immunohistochemistry. From our findings, we report here a P-regulated molecule, Ectonucleotide Pyrophosphatase/ Phosphodiesterase 3 (ENPP3) that can serve as a potential biomarker for progesterone regulated endometrial receptivity. Expression of this glycoprotein was also quantified in uterine fluid with the possible aim to develop a non-invasive endometrial receptivity assay.

## Results

### LCMD and gene expression analysis

**Figure 1:**
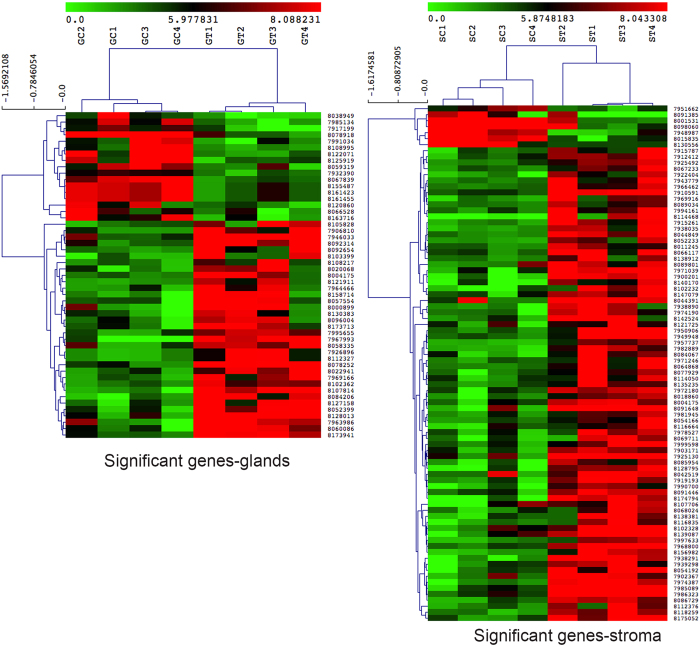
Heatmaps for hierarchical clustering of significant genes. Genes regulated by progesterone in endometrial glandular (G, left panel) and stromal (S, right panel) compartments shown by heatmaps. Gene expression was studied in the receptive endometrium (C) in a non-treated cycle and non-receptive endometrium (T) with the treatment of progesterone receptor antagonist mifepristone. Each woman in the study acted as their own control.

The LCMD of approximately 200 cells yielded a minimum of 500 pg of RNA that was taken for amplification. Microarray data analysis showed 32 genes out of 47 were up-regulated and 15 genes down-regulated in the epithelial compartment, whereas in the stromal compartment, 79 out of 85 genes were up-regulated and 6 down-regulated with mifepristone treatment (Supplementary Table 1). A higher number of significantly downregulated genes were observed in the glands in comparison to stroma with mifepristone treatment (Fig. 1).

### Biological and molecular pathway analysis

The 132 significantly regulated (up or down) genes were analyzed for upstream regulators, canonical pathways, and biological networks. The leading five canonical pathways obtained were NRF2-mediated oxidative stress response, ovarian cancer signaling, superoxide radicals dismutation, prostate cancer signaling, and glioblastoma multiforme signaling, respectively. Ingenuity Pathway Analysis (IPA) predicts activation or inhibition of upstream regulators that may be responsible for gene expression changes observed in the experimental dataset, which aids in understanding the biological activities occurring in the tissues or cells. In the current dataset, transcription factor Chromobox Homolog 5 (CBX5) was predicted to be inhibited with an unbiased Z score of −2.236 and upstream regulator molecules. Colony stimulating factor 2 (CSF2) and early B cell factor 1 (EBF1) were predicted to be activated with a Z scores of 2.43 and 2.0, respectively.

### Validation of microarray by real-time PCR

**Figure 2:**
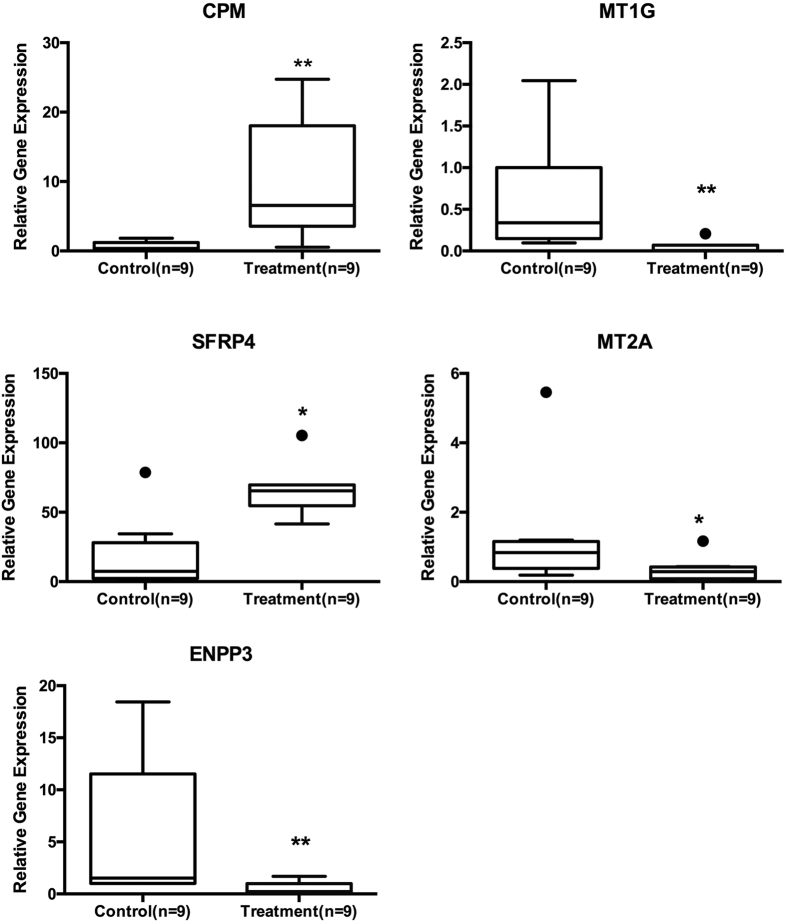
Progesterone regulated genes in endometrial stroma. Tukey plots of significant genes of stromal compartment as analyzed by real time PCR. Progesterone inhibition upregulated CPM and SFRP4 and down regulated MT1G, MT2A and ENPP3.

**Figure 3:**
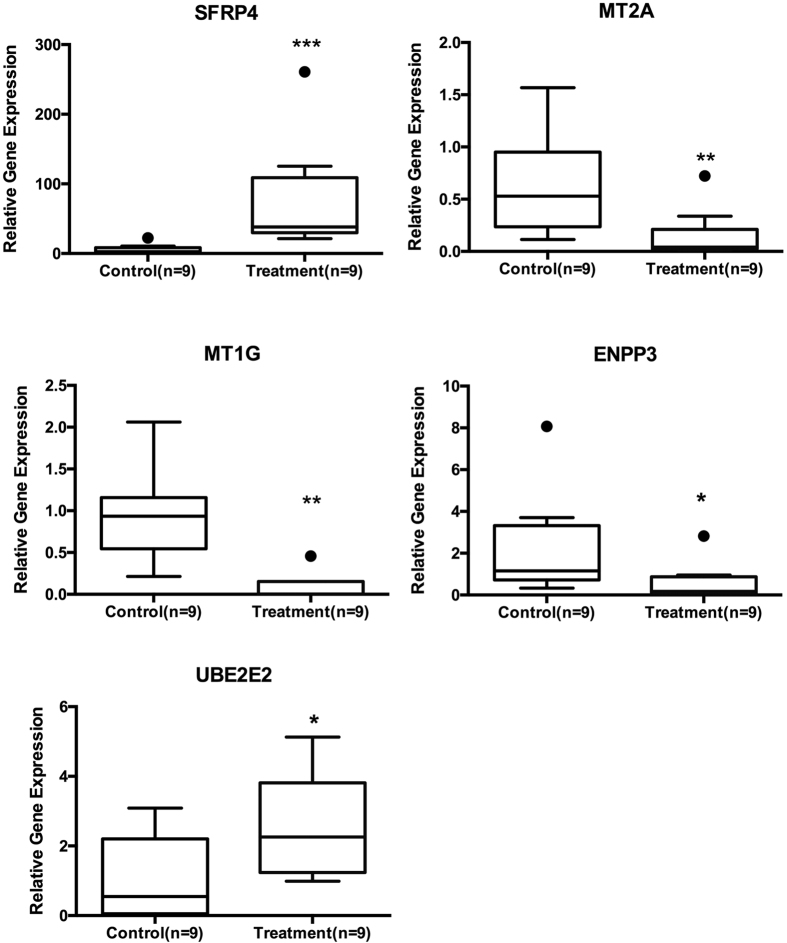
Tukey plot for progesterone regulated genes in endometrial glands. Endometrial epithelial compartment showed down regulation of MT2A, MT1and ENPP3 with the inhibition of P by mifepristone. Gene expression for SFRP4 and UBE2E2 was significantly upregulated with the inhibition of progesterone.

Real-time PCR analysis was in line with the microarray study, as 13 and 11 differentially expressed genes respectively from the stromal and epithelial compartments were corroborated in both methods (supplementary Table 2). Secreted frizzled-related protein 4 (SFRP4) was up regulated by 8.76 fold (p = 0.013) in the stroma compartment and by 16.25 fold (p = 0.0001) in the epithelial compartment. Carboxypeptidase M (CPM) was up-regulated by 29 fold (p = 0.005) in the stromal compartment, while Ubiquitin-Conjugating Enzyme E2E 2 (UBE2E2) was up-regulated by 7 fold (p = 0.024) in the epithelial compartment. MT1G is down-regulated by 7 fold (p = 0.008) in stromal and 519 fold (p = 0.005) in epithelial compartments and MT2A was down-regulated by 3 fold (p = 0.031) in stromal and by 7 fold (p = 0.03) in epithelial compartments. ENPP3 was significantly down-regulated in both the stromal (−55.93 fold; p = 0.015) and epithelial (−9.46 fold; p = 0.035) compartments (Figs 2 and 3).

### Immunohistochemical analysis

**Figure 4:**
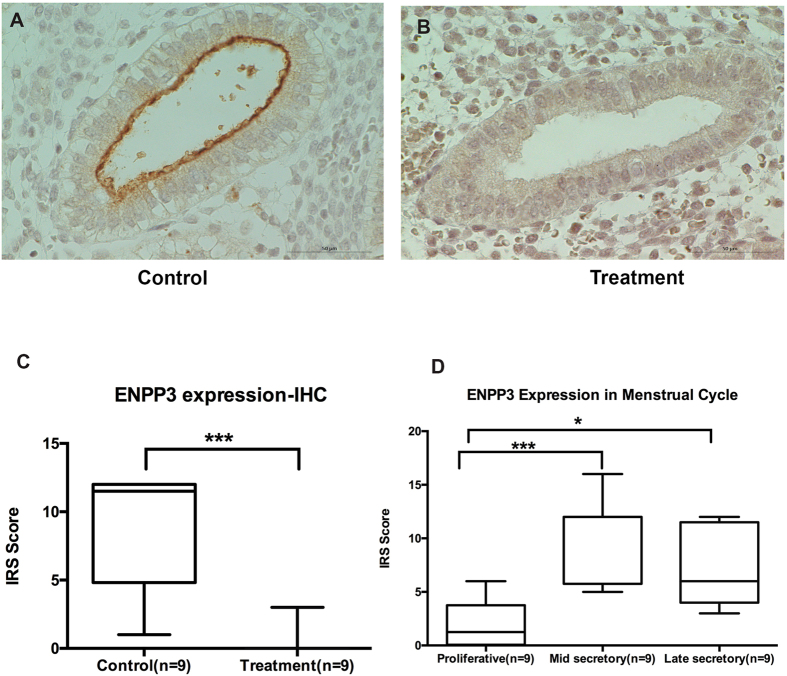
Regulation of ENPP3 by progesterone. The expression of ENPP3 was observed very specifically in the apical border of glands in P dominant mid-luteal phase as studied by immunohistochemistry (**A**,**C**,**D**) in women without mifepristone treatment (control). Downregulation of ENPP3 was observed in endometrial glands with the suppression of progesterone action by mifepristone treatment (**B**,**C**). In endometrium of women without mifepristone treatment, high level of ENPP3 was observed during P upregulated secretory phase, on comparing with proliferative phase of the menstrual cycle. Scale bar indicates 50 μm.

Based on the literature review, microarray expression levels, and availability of antibodies, we studied the protein expression of stanniocalcin 1 (STC1); Cathepsin C (CTSC); Secrteoglobin family 2A member2 (SCGB2A2); SWI/SNF related matrix associated actin-dependent regulator of chromatin subfamily a member 1 (SMARCA1); high-mobility group nucleosome binding domain 5 (HMGN5); and B-cell CLL/lymphoma 11A (zinc finger protein) (BCL11A). Immunodetection of ENPP3 was observed in all the tissues from healthy fertile women and differed significantly between the groups. The findings were in line with the microarray results (Fig. 4A–C).

### Expression of ENPP3 in endometrium

Microarray analysis showed down-regulation of ENPP3 by 59 fold in the epithelial compartment (p = 0.04) with P inhibition. Immunohistochemical analysis for ENPP3 showed expression specific to the apical surface of the epithelial compartment and glandular secretions, but was very scanty in the treatment group. The mean immunoreactive score (IRS ± SD) for the control group was 8.8 ± 4.41 and the treatment group was very low 0.42 ± 1.13 (p = 0.0007). Interestingly, the protein expression for ENPP3 was not seen in stroma in either group, although mRNA expression was seen in both groups as studied by western blot and immunohistochemistry. ENPP3 showed a cyclical expression highest in the mid-secretory phase and lowest in the proliferative phase with mean IRS ± SD scores respectively in proliferative, mid and late-secretory phases were 1.97 ± 2.21, 10.25 ± 3.88, and 7.12 ± 3.83, respectively. Higher expression of ENPP3 was observed in P dominant mid-secretory phase on comparing with the proliferative phase (p = 0.0001). There was no significant difference between mid- and late-secretory phases (Fig. 4D).

### ENPP3 protein expression in uterine fluid and whole endometrial tissue lysates

**Figure 5:**
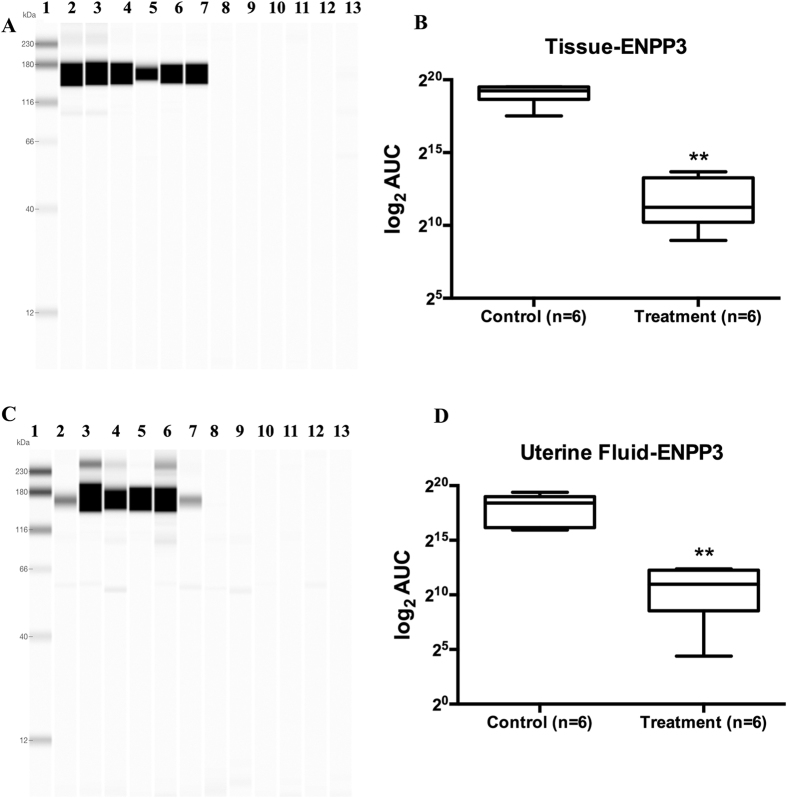
Expression of ENPP3 in endometrium and uterine fluid. Western blot analysis of ENPP3 in receptive phase endometrial tissue lysates (**A**,**B**) and uterine fluid (**C**,**D**) by Wes showed good expression levels of ENPP3 in control samples (lanes 2–7). Antiprogestin treatment (**A**,**C**): lanes 8–13) showed no detectable levels of ENPP3, both in endometrial tissue and uterine fluid, confirming the regulation of ENPP3 protein by progesterone. (**B,D**) show semiquantitative analysis of immunodetectable ENPP3 by Wes, expressed in log_2_ AUC (area under the curve). Lane 1: protein molecular weight marker.

The uterine lavages from fertile women and the same women treated with a single dose of 200 mg of mifepristone on LH + 2 were collected on LH + 6/7 and tested for ENPP3 protein expression by the western blotting technique. A strong band was observed around 165 kd indicating glycosylated ENPP3, and showed a significant downregulation in P-inhibited group (AUC - control 17.85: mifepristone treatment: 9.47; p = 0.002) with a similar pattern to that of tissue ENPP3 (AUC -control 18.98: mifepristone treatment 11.94; p = 0.002). The expression of ENPP3 in endometrial tissue lysate was more abundant than in uterine fluid (Fig. 5).

### Evaluation of ENPP3 as glycoprotein

**Figure 6:**
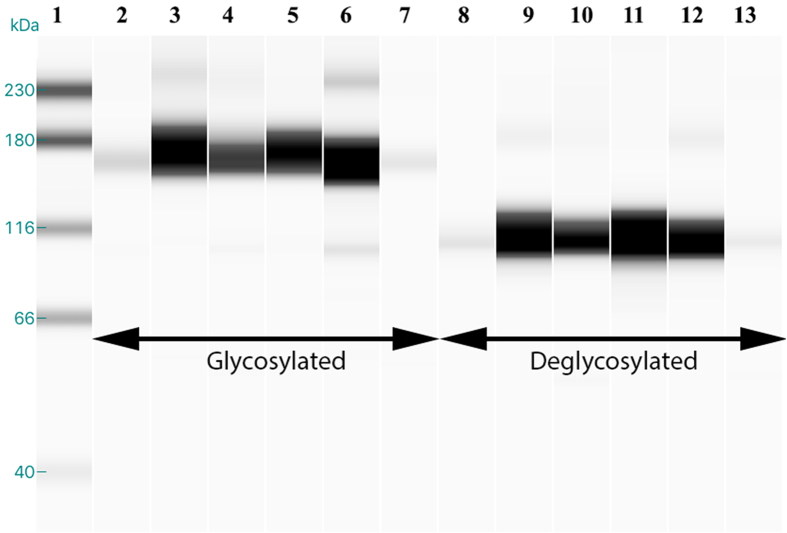
N-glycosylated ENPP3 in human endometrium. Deglycosylation of uterine fluid showed a shift of band from 165 kD to a single band at 110 kD, meaning that ENPP3 in the uterine fluid is present in its glycosylated form. The glycosylated (lanes 2–7) form of ENPP3 showed a band at 165 kD and the deglycosylated (lanes 8–13) form had a single band at 110 kD.

The predicted molecular weight of ENPP3 is around 100 kd, but Western blot analysis showed a band at 165 kd, leading us to further investigate its glycosylation. We tested uterine fluid and endometrial tissue lysates after digesting with Peptide-N-Glycosidase F (PNGase F), which cleaves the glycoaminidase link between asparagine and N-acetylglucosamines. We observed a shift from 165 kD to 110 kD in the deglycosylated samples, confirming that ENPP3 is N-Glycosylated in the uterine fluid and endometria of healthy fertile women (Fig. 6).

### *In vitro* functional assay

**Figure 7:**
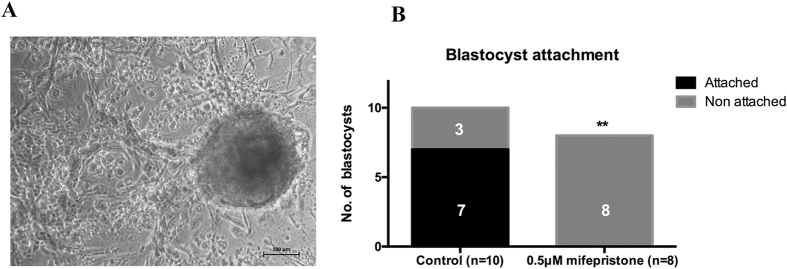
Embryo attachment and ENPP3 expression. Human embryo attached to the *in vitro* three-dimensional cell culture model with progesterone exposure (**A**). None of the 8 embryos in the anti-progestin treated group, where ENPP3 was significantly down regulated had attached. In the control group, 7 out of 10 blastocysts attached (**B**) to the construct, with very good expression of ENPP3 in the three-dimensional endometrial cell culture system.

Embryo attachment was studied in a previously-described three-dimensional endometrial cell culture model to study human implantation process *in vitro*^32^, and showed that 7 out of 10 blastocysts attached to the endometrial construct in the control group. In controls, a very good expression level of ENPP3 was seen as studied by real-time PCR. Exposure to mifepristone (0.5 μM) led to significant (p = 0.004; fisher’s exact test) down-regulation of ENPP3, with none of the embryos attached to the *in vitro* endometrial construct (p = 0.004, Fig. 7).

### Analysis of uterine fluid of receptive and non-receptive phase with nano-ESI-LC/MS/MS

**Figure 8:**
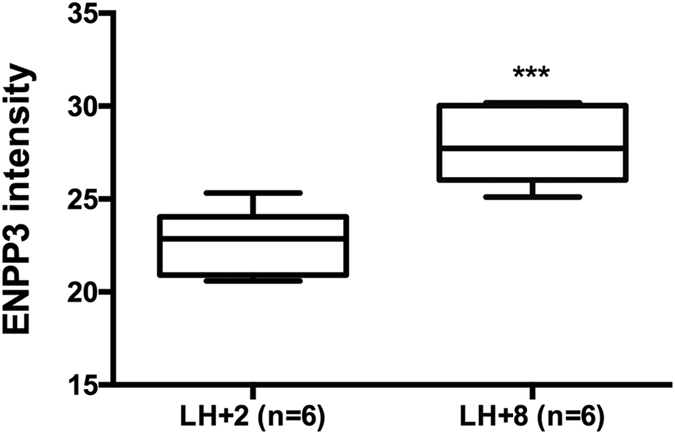
Expression of ENPP3 in the uterine fluid of receptive and non-receptive phase. Tukey plot for the expression of ENPP3 quantified by nano-ESI-LC/MS in uterine fluids from early secretory phase (LH + 2) and mid-secretory phase (LH + 8) of the same set of women. A significant upregulation of ENPP3 (p = 0.0032) was observed in P dominant, receptive phase (LH + 8) on comparison with non-receptive phase (LH + 2).

We identified and quantified the expression of ENPP3 protein with 35 ENPP3 specific peptides obtaining 53.4% sequence coverage in the mass-spectrometry of uterine fluid. Based on total normalized protein intensities, ENPP3 protein was observed to be upregulated (mean fold change + 35.0, p = 0.003) during P dominant LH + 8, on comparing with early secretory phase (LH + 2) of the natural menstrual cycle (Fig. 8).

## Discussion

Our study systematically shows that ENPP3, a N-glycosylated protein regulated by P, is compartmentalized in endometrial glands as well as uterine secretions during the receptive phase. The regulation of ENPP3 by progesterone and the role in implantation process was tested using an *in vitro* human embryo implantation model, where exposure to a P receptor inhibitor inhibited human embryo attachment and thus the embryo implantation process along with the downregulation of ENPP3 expression. As the expression of ENPP3 in the endometrium correlated to that in uterine fluid, we propose that analysis of ENPP3 in uterine fluid may be used as a potential marker in non-invasive test for P regulated endometrial receptivity. Before the use in a clinical setup, we need to validate the data from women with recurrent implantation failure (RIF). This will demand a large number of samples to derive statistical significance to adjust for different factors contributing to RIF.

There are several studies conducted to identify endometrial transcriptome with array technology to explore endometrial receptivity markers with the aim of translating them to clinical use, mainly for diagnosis^20^,^26^,^33^,^34^. None of the above studies, including the studies that compared the mRNA as well as protein profiling between early (LH + 2) and mid (LH + 7 or 8) luteal phase have reported the expression of ENPP3^35^,^36^,^37^. Here, for the first time we report that ENPP3 is highly expressed during progesterone dominant mid-luteal phase (LH + 8) compared with early luteal phase (LH + 2), when the level of P is less. Interestingly, a very recent study conducted to decipher the transcriptome profile of endometrium of RIF patients showed 303 predictive genes and ENPP5, a molecule that belong to ectonucleotide pyrophosphatase family^38^. Though, these reported data are of potential value to identify and develop array based predictive methods^39^,^40^, the functional role of these molecules still remains to be explored and for most of the transcripts, we do not know if they are further transcribed into their protein. In this study, we show the expression of ENPP3 both, at mRNA and protein level in the endometrium and also try to demonstrate its possible role in the process of human embryo implantation with the best available *in vitro* model to study human embryo implantation process. It will be important to further explore the molecular mechanism of ENPP3 in endometrial receptivity and implantation, particularly in female infertility.

Currently, endometrial receptivity array^34^,^39^ (ERA) claims to be a superior and more accurate method for endometrial receptivity evaluation compared to endometrial histological dating using Noyes criteria^41^. However, advanced technology with quality-controlled central laboratories are required for this analyses^42^. A recently developed method based on the expression of integrins^43^ (E-tegrity test^®^) may have potential but needs further clinical studies. It should be noted that both methods are invasive as an endometrial biopsy is required for analysis. The high impact of P on the expression of ENPP3 in the uterine fluid of a receptive endometrium, as reported in this study, is promising and supports its possible use in a receptivity test during the effectual cycle of embryo transfer. Collection of uterine fluid though requires insertion of flexible catheter into the uterine cavity, it does not cause injury to endometrium. More importantly, sampling of uterine fluid in an embryo transfer cycle does not reduce the implantation or pregnancy rate^44^. Hence, we propose that ENPP3 could be used as a marker for a non-invasive test of uterine fluid for progesterone regulated endometrial function. Such tests are not only important in identifying receptive endometrium before starting expensive fertility treatment, but also useful in personalized embryo transfer, that has given the possibility for some women with RIF to conceive^45^.

The novelty of this study compared with that of earlier reports lies in the fact that, we studied the gene expression of major cell types from a cohort of proven fertile women who were their own controls, minimizing the possible wide variations seen in idiopathic infertility. Secondly, stroma and glands in both receptive and non-receptive endometrium were isolated by laser capture microdissection, ruling out the possible global gene expression of different genes seen when using the whole endometrium. Most of the previously-published microarray-based gene expression studies were carried out using whole endometrial tissue^14^,^15^,^20^,^21^,^23^,^46^,^47^, giving the cumulative gene expression effect of all various cell types in the endometrium. We used microarray, a powerful technique to screen vast number of possible genes that are differently expressed. The expression of ENPP3 at transcriptome level was confirmed with a more sensitive and specific method, real time PCR. Further we looked into the expression and distribution of ENPP3 at protein level by Immunohistochemistry. As the expression level of ENPP3 was drastically downregulated with P inhibition, we looked for its expression in the uterine fluid, with the intention of further exploring the possibility of identifying a marker that could be used to develop a less invasive method. For this, we used one of the recently introduced sensitive and rapid method Wes^48^,^49^, that is suitable to analyse low level of protein in very small sample volume as only a very small quantity of protein and uterine fluid could be sampled from women. To study the expression of ENPP3 in receptive and non-receptive phase of the menstrual cycle, we used yet another highly sensitive label free protein analytic technology, nano-ESI-LC/MS/MS^50^. In the past, there are no reports on the functional role of the reported markers on endometrial receptivity as studies in humans are impossible due to ethical reasons. Here, we verified the functional role of ENPP3 in endometrial cells using a previously-described, well-established *in vitro* model for embryo attachment^32^.

The regulation of ENPP3 in endometrial cells is intriguing, as the transcription was downregulated to an extent of 55 times in stromal compartments in non-receptive endometria, but without any detectable levels in protein. This was studied using two different antibodies, both by immunohistochemistry and Western immunoblot. Moreover, the mRNA expression of ENPP3 was more than double in the stromal compartment when compared with the glandular compartment. This high level of mRNA with no protein expression in the stromal compartment for ENPP3 not only reflects post-translational mechanisms at the protein level, but may have a significant biological role, which needs to be studied extensively. In endometrial epithelial cells, ENPP3 is regulated by P, as high levels were observed in the secretory phase of the menstrual cycle in comparison to the proliferative phase. However, we do not see a statistical significance between mid and late secretory phases, though there is a decrease in their mean values. This could be that the threshold level of P required to produce ENPP3 is less and though P is downregulated in the late secretory phase, still a basal level of P exists and this may be enough to produce significant level of ENPP3 that is observed in late luteal phase. In the endometrium, there are reports that ENPP3 is expressed in the epithelial glands as seen by quantitative mass spectrometry analysis in premenopausal women^51^.

We tried to characterize ENPP3, found in the endometrium and uterine fluid. For ENPP3, an immunoblot band is expected around the molecular weight of 100 kd. Surprisingly, a higher molecular weight band around 165 kd was observed, which on further investigation was found to be the glycosylated form present in the uterine fluid samples. We confirmed that this high molecular weight band is the glycosylated form of ENPP3, as treatment of receptive endometrial uterine fluid samples with N-Glycanase resulted in a loss of the immunoblot band at 165 kd and its presence at 100 kd. The expression of ENPP3 in the uterine fluid and endometrial tissue during the receptive phase of the menstrual cycle from proven fertile women had a similar pattern to the glycosylated ENPP3 form. The presence of ENPP3 in glandular secretions as exosomes has been reported earlier as it is present in human parotid glandular secretions encapsulated in exosomes^52^.

ENPP3 is one of the newly described molecules and thus less studied. The expression of ENPP3 in general is mainly reported in basophil and mast cells^53^. ENPP3 is a type II transmembrane protein and lacks the di-leucine that is characteristic of membrane proteins^54^. This is in line with our observation of the endometrium, as ENPP3 is seen only in the apical surface of epithelial cells. The only ENPP family of protein reported in human endometrium is ENPP5, a type I transmembrane protein and there are no report on its function^38^. It would be interesting to study the biological function of ENPP3, especially in relation to its expression and understanding in cases of RIF. One of the challenges in deriving a statistical significance to show the role of ENPP3 in RIF at endometrial level, we may require a large number of samples, as wide range of molecules may contribute individually or collectively to RIF.

The ENPP3 ectoenzyme is involved in extracellular nucleotide hydrolysis and possesses both ATPase and ATP pyrophosphatase activity. It cleaves a variety of phosphodiester and phosphosulfate bonds including deoxynucleotides, nucleotide sugars, and NAD^55^,^56^,^57^. Recently it was shown that ENPP3 regulates the glycosyltransferase activity, which facilitates the glycosylation of many proteins by inhibiting an intrinsic factor for N-acetylglucosaminyltransferase GnT-IX (GnT-Vb) in murine neuroblastoma Neuro 2a cells^58^. Interestingly, the work of Korekane *et al*. shows that ENPP3 can either increase or decrease the activity of glycans^59^, and glycans are present both in the endometrium and uterine secretions, having an important role in the regulation of endometrial receptivity. The expression of Le(Y) oligosaccharide during the receptive period is known to be down-regulated by the inhibition of early luteal phase P by mifepristone in monkey endometria^60^. It is also known that the expression of MUC16 disappears from the endometrium at the time of embryo implantation, coinciding with the period of ENPP3 expression^61^. We do not know the specific glycans regulated by ENPP3 in the endometrium, but this is worth further exploring with regard to its regulation in different gynaecological conditions.

Based on our results we conclude that ENPP3 could be used as a molecular marker for progesterone action at the endometrial level in women. With further studies, this may be used to develop a less invasive method to screen women seeking IVF for endometrial receptivity using uterine fluid, before the planned embryo transfer. On the other hand, it may also be possible to develop an inhibitor or molecule that could alter the function of ENPP3 so it can be used for fertility control. For this, the first step may be to screen the molecular libraries to identify a potent inhibitor. A detailed study to understand the physiological function of this molecule in the endometrium and uterine fluid is essential.

## Methods

### Ethical permissions

All experiments were carried out in accordance with the approved guidelines. The experimental protocols including tissue sampling were approved by Karolinska University Hospital and the regional ethics committee (EPN), Stockholm, Sweden. The Ethical Committee from University of Tartu had approved the permission to collect and perform analysis of uterine fluid collected at their center. Informed and written consent was obtained from all the volunteers participated in the study and the documents were filed as per institutional documentation guidelines.

### Endometrial biopsies

Endometrial biopsies were collected from proven healthy fertile women (n = 9) aged 22–37 years from the upper part of the endometrial cavity with a Pipelle aspirator (Prodimed, Neuilly en Thelle, France). These women were free from any gynaecological disorders and were not taking any hormonal contraception or had any intrauterine devices for a minimum of three months before the biopsy. All the subjects self-examined LH peak in urine samples collected twice a day from approximately cycle day 10 to LH + 2 by using a rapid self-test (Clearplan, Searle Unipath Ltd., Bedford, UK). Biopsies were obtained from each woman on cycle day LH + 7 in a control cycle and thereafter in a treatment cycle. In the treatment cycle, women received a single dose of 200 mg mifepristone on LH + 2. Each biopsy was divided into two portions, with one fixed in 4% paraformaldehyde for immunohistochemistry and the other snap frozen in liquid nitrogen for laser microdissection and RNA extraction. Endometrial samples from healthy women (n = 27) were also obtained during the proliferative, mid-secretory, and late-secretory phases of the menstrual cycle, with nine endometrial samples for each group.

In order to confirm the protein expression of ENPP3 during the receptive phase, 6 endometrial biopsies from control and treatment cycle (200 mg mifepristone on LH + 2) were collected during LH + 6 to LH + 9 from healthy women and were snap frozen in liquid nitrogen till further use. For *in vitro* functional assay by three-dimensional cell cultures, endometrial biopsies were collected from proven fertile women (n = 30) on LH + 4, followed by isolation of stromal and epithelial cells by well-established protocols as previously described^62^.

### Endometrial cell isolation

Briefly, the tissue was minced into 1 × 1 mm in Ham F10 (Life Technologies, Sweden) and incubated with pancreatin-trypsin EDTA (0.05 g/ml of trypsin-EDTA solution) for 30 minutes at 4 °C followed by digestion with Collagenase 4 (125 IU/ml, Worthington Biochemical, Lakewood NJ) and DNAse (40 μg/ml final concentration, Sigma, Sweden) and filtered through a 40-micron mesh cell strainer that allowed stromal cells to pass through and epithelial glands to remain in the strainer. Epithelial glands were digested with collagenase 3 (45 IU/ml, Worthington Biochemical, Lakewood, NJ) and DNAse and filtrated through a 40-micron mesh cell strainer. Stromal and epithelial cells were then separated in a recovery cell culture medium (Life Technologies) and preserved in liquid nitrogen until thawed for the endometrial co-culture.

### Blastocysts

The embryos/blastocysts (n = 18) for this study were acquired through standard *in vitro* fertilization (IVF) or intracytoplasmic sperm injection (ICSI). The embryos used in this study were of at least grade 3BB (Gardner’s classification) and clinically valid for embryo transfer.

### Three-dimensional endometrial cell cultures

Endometrial cell cultures were conducted as described by Lalitkumar *et al*.^63^. After five days of culturing, one group of cultures was treated with mifepristone concentration (Sigma Aldrich) of 0.5 μM (n = 8) and control group was treated with only the vehicle ethanol (n = 10). One embryo was added to each culture on the same day that mifepristone treatment started. The culture medium was changed every two days under light microscope, and cultures were examined for attachment of the embryos. On day five after embryo introduction, attachment rates were noted after mechanical testing by shaking the cultures and washing them with PBS twice. Before termination of cultures, any attached or non-attached embryos were removed. After termination, the cultures were detached from the cell inserts and dissolved in 1 ml of Trizol reagent (Invitrogen) and stored at −80 °C prior to RNA extraction.

### Uterine fluid

Uterine fluid was collected from proven fertile women on LH + 7 with or without mifepristone treatment. Two ml of injection grade water (Gibco) was infused into the uterine cavity using a modified feeding catheter (Nutrisafe 2, Fr-L.75 cm, Vycon Value Life, Ecouen, France) fixed to a 10 ml syringe. With the help of the syringe, about 1 ml was sucked back along with the uterine lavage, and centrifuged at 200 g for 10 minutes to remove any cells or cell debris. The supernatant was lyophilized and reconstituted in 50 μl sterile water and taken for further analysis.

In another set of fertile women (n = 6; University of Tartu, Estonia), uterine fluid was collected in the same cycle, during early secretory phase (LH + 2) and progesterone dominant mid-secretory (LH + 8) phase to perform label-free proteomic analysis for ENPP3 expression.

### Automated western blot

A completely automated western blot was performed using Simple Wes (Protein Simple, San Jose, CA) as per the manufacturer’s instructions. Briefly, 2 μg of protein from the tissue lysates or uterine fluid was added to the standard fluorescent mastermix and loaded into corresponding wells of the prefilled Wes assay plate, along with antibody diluent (Protein Simple), anti-ENPP3 antibody (1:50 dilution Sigma LifeSciences, HPA043772), anti-rabbit secondary antibody (Protein Simple), and Streptavidin, followed by luminol peroxide mix. The imaging and analysis were done with compass software (Protein Simple).

### Label-free proteomic analysis by Nano-ESI-LC/MS/MS

The uterine fluids were separated into six fractions based on its molecular weight using SDS-PAGE (Invitrogen). Proteins were then reduced, alkylated and in-gel digested with dimethylated porcine trypsin (Sigma) followed by analysis with nano-ESI-LC/MS/MS (Dionex Ultimate 3000 RSLC and Q Exactive MS/MS, Thermo Fisher Scientific at University of Tartu) using 2 h reversed phase gradients and a top-10 data-dependent acquisition method. The obtained label-free mass-spectrometric data were identified and quantified with MaxQuant software package^64^ (UniProtKB human reference proteome database, 2014 September version). Label-free data were normalized with the MaxLFQ algorithm^50^ and compared with paired or unpaired two-tailed t-test.

### Fluorescence-activated cell sorting (FACS)

Stromal cells were selectively isolated from the pool of endometrial cells by negative selection with FACS. Briefly, the cells were washed and stained for epithelial cells with EPCAM/CD326 for 30 minutes and were sorted using MoFLOW^®^ XDP flow cell activated cell sorter (Beckman Coulter, USA).

### Immunohistochemistry

The 5 μm paraffin-embedded endometrial tissue samples were deparaffinized using a 2100-retriever autoclave (Histolab, Gothenburg, Sweden) and antigen retrieval done with a Diva Decloaker (Biocare Medical, Concord, CA). After peroxidase quenching, the sections were incubated with Background Sniper (Biocare Medical) followed by incubation with the primary antibodies (Supplementary Table 3) overnight at 4 °C. The primary antibodies were diluted in the diluent DaVinci Green (Biocare Medical, Concord, CA). The biotin-free detecting system Rabbit/Mouse HRP polymer kit MACH 3^TM^ (Biocare Medical, Concord, CA) was used for detection. The reaction was developed with the Betazoid DAB Chromogen kit (Biocare Medical, Concord, CA). The counterstained hematoxylin (Vector Laboratories, Inc., Burlingame, CA) sections were mounted using the xylene-based medium Pertex^®^ (Histolab, Gothenburg, Sweden) and analyzed in a Zeiss Axiovert 200 M microscope (Zeiss, Göttingen, Germany)^62^. All the slides were blinded and scored by two independent observers. The semi-quantitative IRS-score^65^ was used to assess the percentage of the positive cells (PC) and the staining intensity (SI). On disparity between the two observers, a third independent observer analysed the blinded slides and the average score of the two closest results was taken for further analysis.

### Deglycosylation

Endometrial tissue lysates and uterine fluid samples were subjected to mild denaturation with 0.2% Rapigest SF Surfactant (Waters, 186001861) and DTT (5 mM) and the samples were denatured for 5 minutes at 95 °C. Iodoacetamide (15 mM final concentration) was added and incubated for 30 minutes in dark, and in-solution enzymatic digestion was done by incubation with 1 μl/sample PNGase F (Roche, 11365169001) for 2 hours at 37 °C and analyzed by Simple Wes.

### Statistical analysis

The paired T-test and SAM methods were performed to analyze microarray data by MultiExperiment Viewer. The Mann-Whitney U test was applied to compare immunohistochemical staining between the control and treatment groups. Based on the statistical assumptions, either an unpaired T-test or Mann Whitney test was performed for analyzing the real time PCR and western blot data. Fisher’s exact test was performed to analyze blastocyst attachment rate. All the statistical analysis was done with XLSTAT 2015 (Addinsoft) and Prism 6 (GraphPad Software).

## Additional Information

**Accession code**: Microarray data, GEO repository accession number GSE59447.

**How to cite this article**: Boggavarapu, N. R. *et al*. Compartmentalized gene expression profiling of receptive endometrium reveals progesterone regulated ENPP3 is differentially expressed and secreted in glycosylated form. *Sci. Rep.* **6**, 33811; doi: 10.1038/srep33811 (2016).

## Supplementary Material

Supplementary Information
