# Supplementary material for: Compartmentalized gene expression profiling of receptive endometrium reveals progesterone regulated ENPP3 is differentially expressed and secreted in glycosylated form
Source: Sci Rep. 2016 Sep 26;6:33811. doi: 10.1038/srep33811 (PMC5036034; doi:10.1038/srep33811)
Supplement: Supplementary Information [file srep33811-s1.pdf]

Supplementary material

Compartmentalized gene expression profiling of receptive endometrium reveals progesterone regulated ENPP3 is differentially expressed and secreted in glycosylated form.

Nageswara Rao Boggavarapu[#], Sujata Lalitkumar[#], Vijay Joshua[#], Sergo Kasvandik[§], Andres Salumets[§], Parameswaran Grace Lalitkumar[#,*], Kristina Gemzell Danielsson[#,*]

[#] Division of Obstetrics and Gynecology, Department of Women's and Children's Health, Karolinska Institutet/ Karolinska University Hospital, S-171 76, Stockholm, Sweden.

[§] Competence Centre on Health Technologies, Tiigi 61b, 50410, Tartu, Estonia; Department of Obstetrics and Gynecology, University of Tartu, L. Puusepa 8, 51014, Tartu, Estonia; Department of Obstetrics and Gynecology, Helsinki University Hospital, Haartmaninkatu 2, 00290, Helsinki, Finland

Methods- Supplementary material

Laser capture microdissection (LCMD)

Frozen endometrial tissues were sectioned at 10 µm using a cryotome (Reichert Jung Cryocut 1800, Leica) and fixed onto membrane slides (Membrane slide NF 1.0 PEN, Carl Zeiss Microimaging GmbH, Germany) with 70% ethanol at -20°C for 30 minutes. Prior to use, the membrane slides were made hydrophilic by UV irradiation for 30 minutes. The tissue sections were stained using Histogene® staining solution (Applied Biosystems, Life Technologies, Carlsbad, CA) and serially dehydrated and stored at -80°C till they were used for laser dissection.

Epithelial glands and stromal cells were dissected out using LCM using a PALM Laser-Microbeam system and PALM Robosoftware (Carl Zeiss Microimaging GmbH, Germany) with a pulsed 355 nm diode laser to cut and catapult the sample onto a cup of 0.5ml MicroTube 500 (Carl Zeiss Microimaging GmbH, Germany) containing 35 µL of extraction buffer (Arcturus® Picopure® Frozen RNA Isolation kit, Applied Biosystems, Life Technologies, Carlsbad, CA). Approximately 200 cells were dissected from each section, briefly centrifuged, and stored at -80°C until further use. All the above-mentioned steps were performed in a strictly RNAse-free environment to maintain RNA integrity for microarray hybridizations. A separate set of dissections was carried out for real-time PCR analysis.

RNA extraction and amplification

RNA was extracted from LCMD samples using an Arcturus® Picopure® Frozen RNA Isolation kit as per the manufacturer's protocol and subjected to DNAse treatment to eliminate genomic DNA if any. Quality control and concentration of RNA was performed using Agilent RNA 6000 Pico Kit and Agilent 2100 Bioanalyzer (Agilent Technologies Inc., Santa Clara, CA). Samples with an RNA yield of >500 pg and a quality of 28S:18S > 1.5 and RIN > 7.5 were used for microarray and real-time PCR. RNA amplification was performed with NUGEN Ovation Pico WTA System™ (Nugen Technologies Inc., San Carlos, CA). RNA extraction from trizol-lyzed, three-dimensional cell cultures was performed with purelink RNA micro kit (Life Technologies, Sweden).

Microarray

Microarray (n=8 each, paired samples) was performed at the Core Facility for Bioinformatics and Expression Analysis located at the Department of Biosciences and Nutrition, Karolinska Institutet. The amplified complimentary mRNA was used to generate the sense target of cDNA using NuGen WT-Ovation™ Exon Module version 1.0 (Nugen Technologies Inc., San Carlos, CA). Fragmentation, biotin labeling, and target preparation of the ST cDNA were performed using the NuGEN EncoreTM Biotin Module (NuGEN Technologies Inc., San Carlos, CA). Hybridization of ST cDNA was performed on the Affymetrix GeneChip® Human Gene 1.0 ST array (Affymetrix, Santa Clara, CA). This array had 764,885 probes that correspond to 28,829 well-annotated genes. The CEL files from microarray were processed using Affymetrix Expression Console™ software, with expression data obtained for all samples.

Microarray data analysis

The initial analysis of the data was performed with Affymetrix Expression Console™ software. The summarization algorithm used was Iterative Probe Logarithmic Intensity Error Estimation (IterPLIER). The advantage of IterPlier is that it performs PLIER estimation and iteratively discards the non-correlating probe sets. Background correction was done using GC-Composition-based background correction (PM-GCBG). Normalization was performed by the quantile sketch method and the data was log2-transformed after extraction from the console software. Probesets that were not annotated in the database were discarded; only annotated genes were considered for further analysis.

The data was tested for any interfering outliers using R software, and the data was filtered to remove background and noise before any statistical analysis was performed. Gene probes with median expression intensities less than 6 in either the control or treatment group were removed. Fold change was calculated for each gene probe and only those genes that were up or downregulated by two times in all pairs were used for further statistical analysis. Differentially-regulated significant genes were selected from a paired T-Test with Welch approximation, and P values based on T-distribution and fold change for each significant gene were calculated by a significance analysis of microarrays method (SAM) in Multiexperiment Viewer (MeV, part of TM4 Microarray software suite). The significant gene list obtained from this analysis was further analyzed for functional and canonical pathways by

using Ingenuity Pathway Analysis (IPA) (Ingenuity System, www.ingenuity.com) software. IPA is a web-based application that performs analysis, integration, and interpretation of the given data using its own comprehensive, manually-curated content of the ingenuity knowledge base. This knowledge base includes information from various sources like Entrez gene, RefSeq, OMIM, GWAS, and Gene Ontology.

Real-time PCR

Real-time PCR was performed using a StepOne Plus instrument (Applied Biosystems). TaqMan Universal PCR Master Mix (Applied Biosystems) was used in a microamp fast optical 96 well reaction plate with a relative quantification method. 18S was used as a reference gene and samples were run in triplicate. Real-time PCR was run according to the manufacturer's instructions with an equal quantity of cDNA in all the samples. Real-time PCR data was analyzed using StepOne Plus software (Applied Biosystems).

Supplementary Table 1:
List of differentially regulated genes in endometrial epithelial compartment with the downregulation of progesterone receptor action by mifepristone

ProbesetID	Gene Symbol	Gene Description	Foldchange
8078918	SNORA62	small nucleolar RNA, H/ACA box 62	-65.62
8122071	ENPP3	ectonucleotide pyrophosphatase/ phosphodiesterase 3	-59.01
7985134	IDH3A	isocitrate dehydrogenase 3 (NAD+) alpha	-39.1
8163716	BRINP1	bone morphogenetic protein/retinoic acid inducible neural-specific 1	-38.88
8038949	ZNF841	zinc finger protein 841	-34.53
8067839	FGF7P2	fibroblast growth factor 7 pseudogene 2	-26.55
7917199	TTLL7	tubulin tyrosine ligase-like family, member 7	-23.66
8125919	FKBP5	FK506 binding protein 5	-19.93
7991034	HOMER2	homer homolog 2 (Drosophila)	-19.81
8155487	FGF7	fibroblast growth factor 7	-17.62
8108995	SCGB3A2	secretoglobin, family 3A, member 2	-15.25
8059319	FARSB	phenylalanyl-tRNA synthetase, beta subunit	-13.46
8066528	PIGT	phosphatidylinositol glycan anchor biosynthesis, class T	-12.48
7932390	TRDMT1	tRNA aspartic acid methyltransferase 1	-9.05
8120860	BCKDHB	branched chain keto acid dehydrogenase E1, beta polypeptide	-8.24
8105828	CCNB1	cyclin B1	10.32
8108217	TGFBI	transforming growth factor, beta-induced, 68kDa	10.43
7964466	DCTN2	dynactin 2 (p50)	11.76
8130383	MTRF1L	mitochondrial translational release factor 1-like	12.79
8121911	CENPW	centromere protein W	12.97
8058335	SUMO1	small ubiquitin-like modifier 1	14.26
8004175	TXNDC17	thioredoxin domain containing 17	14.59
7995655	FTO	fat mass and obesity associated	15.4
8092654	RPL39L	ribosomal protein L39-like	16.25
8102362	TIFA	TRAF-interacting protein with forkhead-associated domain	16.41
8022941	TPGS2	tubulin polyglutamylase complex subunit 2	16.65
8020068	ANKRD12	ankyrin repeat domain 12	17.49
8103399	PDGFC	platelet derived growth factor C	18.59
8173713	MAGT1	magnesium transporter 1	19.5
8173941	TSPAN6	tetraspanin 6	19.98
7906810	DUSP12	dual specificity phosphatase 12	20.14
8078252	UBE2E2	ubiquitin-conjugating enzyme E2E 2	20.64
7969166	KCNRG	potassium channel regulator	22.19
8060086	MYEOV2	myeloma overexpressed 2	22.51
8127158	GCLC	glutamate-cysteine ligase, catalytic subunit	23.63

8092314	DNAJC19	DnaJ (Hsp40) homolog, subfamily C, member 19	24.93
8052399	BCL11A	B-cell CLL/lymphoma 11A (zinc finger protein)	27.11
8128013	RARS2	arginyl-tRNA synthetase 2, mitochondrial	29.32
8112327	CKS1B	CDC28 protein kinase regulatory subunit 1B	34.64
7963986	RAB13	RAB13, member RAS oncogene family	39.78
8158714	EXOSC2	exosome component 2	41.02
7946033	HBB	hemoglobin, beta	45.24
8057554	CACYBP	calcyclin binding protein	45.55
8000899	ZNF768	zinc finger protein 768	71.2
8084206	B3GNT5	UDP-GlcNAc:betaGal beta-1,3-N-acetylglucosaminyltransferase 5	80.51
8107814	ISOC1	isochorismatase domain containing 1	131.81
7967993	FGF9	fibroblast growth factor 9	854.95

Significant gene list in Stromal compartment

ProbesetID	Gene Symbol	Gene Description	Fold change
8001531	MT1G	metallothionein 1G	-69.92
7948987	PLA2G16	phospholipase A2, group XVI	-45.77
8098060	RXFP1	relaxin/insulin-like family peptide receptor 1	-39.16
8015835	DUSP3	dual specificity phosphatase 3	-35.21
8130556	SOD2	superoxide dismutase 2, mitochondrial	-20.98
7951662	CRYAB	crystallin, alpha B	-9.23
8089034	ARMC10	armadillo repeat containing 10	8.98
8138912	LSM5	LSM5 homolog, U6 small nuclear RNA associated (S. cerevisiae)	9.87
7981945	SNRPN	small nuclear ribonucleoprotein polypeptide N	9.88
8068024	JAM2	junctional adhesion molecule 2	10.25
8114050	Sep-08	septin 8	10.61
7915787	PIK3R3	phosphoinositide-3-kinase, regulatory subunit 3 (gamma)	10.96
8011245	TSR1	TSR1, 20S rRNA accumulation, homolog (S. cerevisiae)	10.99
8054166	TSGA10	testis specific, 10	11.4
8112376	CENPK	centromere protein K	11.45
7978527	DTD2	D-tyrosyl-tRNA deacylase 2 (putative)	11.58
8135235	PMPCB	peptidase (mitochondrial processing) beta	12
8116664	PRPF4B	pre-mRNA processing factor 4B	12.43
8067233	PMEPA1	prostate transmembrane protein, androgen induced 1	12.69
8089801	GSK3B	glycogen synthase kinase 3 beta	13.26
8147079	LRRCC1	leucine rich repeat and coiled-coil centrosomal protein 1	13.54
7925492	OPN3	opsin 3	13.67
7971246	VWA8	von Willebrand factor A domain containing 8	13.96

7994161	RBBP6	retinoblastoma binding protein 6	14.54
8004175	TXNDC17	thioredoxin domain containing 17	14.59
8052233	CLHC1	clathrin heavy chain linker domain containing 1	14.65
7943779	C11orf1	chromosome 11 open reading frame 1	14.72
7999598	NTAN1	N-terminal asparagine amidase	14.87
7903171	RWDD3	RWD domain containing 3	15.91
8121725	BRD7P3	bromodomain containing 7 pseudogene 3	16.04
7912412	MTOR	mechanistic target of rapamycin (serine/threonine kinase)	16.58
7966462	NAA25	N(alpha)-acetyltransferase 25, NatB auxiliary subunit	16.67
7902367	ACADM	acyl-CoA dehydrogenase, C-4 to C-12 straight chain	16.75
8142524	TSPAN12	tetraspanin 12	16.83
8091446	PFN2	profilin 2	17.22
8042519	PCYOX1	prenylcysteine oxidase 1	17.31
8069711	LTN1	listerin E3 ubiquitin protein ligase 1	17.33
8116835	GCNT2	glucosaminyl (N-acetyl) transferase 2, I-branching enzyme (I blood group)	17.72
7949948	C11orf24	chromosome 11 open reading frame 24	17.94
7997633	USP10	ubiquitin specific peptidase 10	18.2
7969916	BIVM	basic, immunoglobulin-like variable motif containing	18.29
7972180	RNF219	ring finger protein 219	19.34
8064868	GPCPD1	glycerophosphocholine phosphodiesterase GDE1 homolog (S. cerevisiae)	19.61
8066117	SAMHD1	SAM domain and HD domain 1	20.79
7910591	NTPCR	nucleoside-triphosphatase, cancer-related	20.96
8018860	BIRC5	baculoviral IAP repeat containing 5	22.32
8086729	KIF9	kinesin family member 9	23.38
8102328	CFI	complement factor I	23.4
8138381	AGR2	anterior gradient 2	23.6
7925130	TARBP1	TAR (HIV-1) RNA binding protein 1	23.81
8044391	MERTK	MER proto-oncogene, tyrosine kinase	24.68
7957737	TMPO	thymopoietin	24.74
8077929	RNU6-377P	RNA, U6 small nuclear 377, pseudogene	25.69
7922404	SNORD80	small nucleolar RNA, C/D box 80	25.75
7915261	TRIT1	tRNA isopentenyltransferase 1	28.46
7939298	CAT	catalase	29.1
8107706	LMNB1	lamin B1	29.24
7950906	CTSC	cathepsin C	29.39
7968800	DGKH	diacylglycerol kinase, eta	29.84
8084067	ACTL6A	actin-like 6A	30.15
7938035	TRIM22	tripartite motif containing 22	30.32

8054192	MITD1	MIT, microtubule interacting and transport, domain containing 1	30.7
8044849	PTPN4	protein tyrosine phosphatase, non-receptor type 4 (megakaryocyte)	31.52
7919193	NUDT4	nudix (nucleoside diphosphate linked moiety X)-type motif 4	32.43
8085954	AZI2	5-azacytidine induced 2	33.14
8175052	AIFM1	apoptosis-inducing factor, mitochondrion-associated, 1	34.41
8118259	MSH5	mutS homolog 5	34.52
7985089	RCN2	reticulocalbin 2, EF-hand calcium binding domain	38.88
7971039	SUPT20H	suppressor of Ty 20 homolog (S. cerevisiae)	40.52
7982889	NUSAP1	nucleolar and spindle associated protein 1	41.43
7986323	LOC440311	glioma tumor suppressor candidate region gene 2 pseudogene	44.19
8156982	SMC2	structural maintenance of chromosomes 2	45.66
7990700	WDR61	WD repeat domain 61	63.4
8140170	GTF2IRD2B	GTF2I repeat domain containing 2B	64.02
8139087	SFRP4	secreted frizzled-related protein 4	67.49
8091648	SSR3	signal sequence receptor, gamma (translocon-associated protein gamma)	70.96
7974190	DNAJC19	DnaJ (Hsp40) homolog, subfamily C, member 19	73.32
7938890	PRMT3	protein arginine methyltransferase 3	87.47
7974387	STYX	serine/threonine/tyrosine interacting protein	88.2
7900201	UTP11L	UTP11-like, U3 small nucleolar ribonucleoprotein (yeast)	127.29
8128795	AK9	adenylate kinase 9	186.43
8102232	LEF1	lymphoid enhancer-binding factor 1	215.96
7938291	SNORA45A	small nucleolar RNA, H/ACA box 45A	352.12
8114468	SNORD63	small nucleolar RNA, C/D box 63	441.38
8174794	CUL4B	cullin 4B	652.03

Supplementary Table 2: Validation of microarray results of selected genes that were differentially regulated with P inhibition by Real time PCR both in the endometrial glandular and stromal compartments

Glandular Compartment							
**Gene**	**Control ΔCт Mean**	**Control ΔCт SE**	**Treatment ΔCт Mean**	**Treatment ΔCт SE**	**ΔΔCт**	**Fold Change**	**P Value**
MT1G	3.648	0.055	12.669	0.261	9.021	-519.60	**0.005**
ENPP3	3.928	0.098	7.171	0.065	3.243	-9.46	**0.015**
MT2A	0.843	0.022	3.654	0.026	2.811	-7.02	**0.003**
UBE2E2	11.565	0.073	8.747	0.055	-2.818	7.052	**0.024**
SFRP4	3.635	0.072	-0.388	0.072	-4.022	16.251	**0.0001**
RPL27A	10.647	0.128	8.556	0.067	-2.091	4.262	0.095
BCL11A	10.940	0.097	7.887	0.076	-3.054	8.302	0.132
POSTN	9.893	0.112	8.467	0.096	-1.425	2.686	0.352
STC1	9.445	0.169	11.904	0.116	2.459	-5.50	0.403
RHOJ	7.755	0.108	7.190	0.072	-0.565	1.480	0.621
VPS53	8.420	0.086	8.318	0.059	-0.102	1.073	0.942
**Stromal Compartment**							
**Gene**	**Control ΔCт Mean**	**Control ΔCт SE**	**Treatment ΔCт Mean**	**Treatment ΔCт SE**	**ΔΔCт**	**Fold Change**	**P Value**
MT1G	3.442	0.063	10.525	0.118	7.083	-135.55	**0.008**
ENPP3	4.567	0.109	10.372	0.109	5.805	-55.93	**0.035**
MT2A	0.880	0.042	2.694	0.037	1.813	-3.51	**0.031**
SFRP4	0.748	0.079	-2.385	0.082	-3.132	8.769	**0.013**
CPM	12.638	0.147	7.777	0.107	-4.861	29.055	**0.005**
SOD2	3.469	0.069	4.027	0.047	0.558	-1.47	0.340
BCL11A	6.212	0.092	6.489	0.080	0.277	-1.21	0.378
ATP6V0E1	6.632	0.087	6.280	0.095	-0.353	1.277	0.227
HMGN5	6.684	0.061	6.811	0.033	0.127	-1.09	0.852
SNORA3	9.915	0.087	10.257	0.169	0.342	-1.27	0.889
RPL27A	8.655	0.060	8.483	0.064	-0.172	1.127	0.900
SMARCA1	5.564	0.071	4.892	0.047	-0.672	1.593	0.958
CTSC	8.060	0.086	7.065	0.059	-0.995	1.993	0.198

Supplementary Table 3:

Details of antibodies used for immunohistochemcial Analysis

Antibody	Catalogue No.	Dilution used	Manufacturer
anti-STC1	SC-30183	1:100	Santa Cruz Biotechnologies
anti-CTSC	SC-74590	1:400	Santa Cruz Biotechnologies
anti-SMARCA1	PAB20193	1:50	Abnova
anti-SCGB2A2	MAB10386	1:25	Abnova
anti-BCL11A	H00053335	1:150	Abnova
anti-NSBP1	AB56031	1:200	Abcam
anti-ENPP3	HPA043772	1:500	Sigma Aldrich
